# Safety, feasibility, tolerability, and clinical effects of repeated psilocybin dosing combined with non-directive support in the treatment of obsessive-compulsive disorder: protocol for a randomized, waitlist-controlled trial with blinded ratings

**DOI:** 10.3389/fpsyt.2023.1278823

**Published:** 2024-01-09

**Authors:** Terence H. W. Ching, Lucia Amoroso, Calvin Bohner, Elizabeth D’Amico, Jeffrey Eilbott, Tara Entezar, Madison Fitzpatrick, Geena Fram, Rachael Grazioplene, Jamila Hokanson, Anastasia Jankovsky, Stephen A. Kichuk, Bradford Martins, Prerana Patel, Henry Schaer, Sarah Shnayder, Chelsea Witherow, Christopher Pittenger, Benjamin Kelmendi

**Affiliations:** ^1^Department of Psychiatry, Yale University School of Medicine, New Haven, CT, United States; ^2^Department of Psychology, Yale University, New Haven, CT, United States; ^3^Center for Brain and Mind Health, Yale University School of Medicine, New Haven, CT, United States; ^4^Child Study Center, Yale University School of Medicine, New Haven, CT, United States

**Keywords:** obsessive-compulsive disorder, psilocybin, psychedelic, non-directive support, waitlist, treatment, mental health, adult psychiatry

## Abstract

**Background:**

To date, few randomized controlled trials of psilocybin with non-directive support exist for obsessive-compulsive disorder (OCD). Results and participant feedback from an interim analysis of an ongoing single-dose trial (NCT03356483) converged on the possibility of administering a higher fixed dose and/or more doses of psilocybin in future trials for presumably greater benefits.

**Objectives:**

This trial aims to evaluate the safety, feasibility, tolerability, and clinical effects of two doses of psilocybin paired with non-directive support in the treatment of OCD. This trial also seeks to examine whether two doses of psilocybin lead to greater OCD symptom reduction than a single dose, and to elucidate psychological mechanisms underlying the effects of psilocybin on OCD.

**Design:**

A randomized (1:1), waitlist-controlled design with blinded ratings will be used to examine the effects of two doses of oral psilocybin paired with non-directive support vs. waitlist control on OCD symptoms. An adaptive dose selection strategy will be implemented (i.e., first dose: 25 mg; second dose: 25 or 30 mg).

**Methods and analysis:**

This single-site trial will enroll 30 adult participants with treatment-refractory OCD. Aside from safety, feasibility, and tolerability metrics, primary outcomes include OCD symptoms assessed on the Yale-Brown Obsessive-Compulsive Scale – Second Edition (Y-BOCS-II). A blinded independent rater will assess primary outcomes at baseline and the primary endpoint at the end of the second dosing week. Participants will be followed up to 12 months post-second dosing. Participants randomized to waitlist will be rescreened after 7 weeks post-randomization, and begin their delayed treatment phase thereafter if still eligible.

**Ethics:**

Written informed consent will be obtained from participants. The institutional review board has approved this trial (protocol v. 1.7; HIC #2000032623).

**Discussion:**

This study seeks to advance our ability to treat refractory OCD, and catalyze future research seeking to optimize the process of psilocybin treatment for OCD through understanding relevant psychological mechanisms.

**Clinical trial registration**: ClinicalTrials.gov, identifier NCT05370911.

## 1 Introduction

### 1.1 Background and rationale

Obsessive-compulsive disorder (OCD) is characterized by obsessions (i.e., recurrent, intrusive, anxiety-provoking thoughts, images, or impulses) and compulsions (i.e., repetitive and/or ritualized behaviors performed in an attempt to relieve anxiety or distress). OCD has a lifetime prevalence rate of 2.3% among adults in the U.S. (1), and can often be chronically disabling (2–4), in part due to significant delays in treatment (5).

First-line treatments for OCD include selective serotonin reuptake inhibitors (SSRIs) (6) and cognitive-behavior therapy with exposure and response prevention (CBT/ERP) (7–10). These treatments, however, have long-standing limitations. A significant proportion (up to 60%) of patients are non-responsive to SSRI trials (11–14), and patients may continue to lead restricted lives even after completing a course of CBT/ERP (15). Relapse is also common after discontinuing or completing these treatments (16, 17). Additionally, there is limited access to clinicians who can competently deliver CBT/ERP (18–22). Furthermore, step-up care options can be demanding, including SSRI augmentation (23), residential treatment (24), or even neurosurgery for treatment-refractory OCD (25). Therefore, additional innovative, faster-acting, and minimally invasive treatment options are needed.

Psilocybin (4-phosphryloxy-N,N-dimethyltryptamine) is found naturally in certain mushrooms, but is typically administered in controlled settings in a pure, synthetic, encapsulated form. When ingested, psilocybin is metabolized in the intestinal mucosa to the psychoactive metabolite psilocin, which acts as a high-affinity serotonin agonist, leading to various downstream effects (26, 27). Acute perceptual effects of psilocin action include altered perception of time, space, and motion, as well as paresthesia (28). Other acute psychological effects can include altered information processing, dizziness, impaired concentration, depersonalization, derealization, and significant emotions or mood changes (28, 29). Psilocin also tends to activate the sympathetic nervous system, leading to body temperature changes and increased blood pressure and heart rate (30). Other common physical adverse effects include mild headache, fever, fatigue, nausea, diarrhea, dizziness, and mild lethargy. These effects tend to be transient, resolving typically after 6 hours from ingestion (28, 30). Psilocybin is also completely eliminated from the body within 24 h in healthy participants (31). Further, the safety profile of psilocybin in controlled research settings is robust; participants tend to navigate these acute experiences without the need for medical intervention, and rates of serious adverse events (SAEs) are low in these settings (32, 33). Although hallucinogen persisting perception disorder (HPPD) has been viewed as an adverse event of special interest (AESI) in psychedelic research, no convincing evidence has been found for HPPD symptoms with controlled administrations of psilocybin.

Psilocybin dosing combined with psychological support has been examined as a promising treatment option in clinical trials for various disorders, including treatment-resistant depression, psychological distress associated with a cancer diagnosis, and problematic alcohol and tobacco use (34). An open-label trial of single-dose psilocybin paired with psychological support for 12 individuals with body dysmorphic disorder (BDD) – a condition on the OC and related disorders (OCRD) spectrum – was also recently completed, in which 58% of the sample reported persisting clinically significant BDD symptom improvement at 12-week follow-up (35).

Of interest, there has been only one study of psilocybin completed in individuals with OCD (36). In this proof-of-concept, pilot study of nine treatment-refractory participants with OCD, repeated psilocybin dosing (i.e., 100, 200, then 300 μg/kg, with a double-blind 25 μg/kg dose randomly inserted) paired with unstructured psychological support was shown to be safe, tolerable, and feasible. No SAEs were reported, except for an instance of transient hypertension in one participant. In terms of clinical effects, there was a 23 to 100% reduction in OCD symptom severity 24 h after dosing across the sample, as measured on the Yale-Brown Obsessive-Compulsive Scale (Y-BOCS) (37, 38). Additionally, one participant continued to be in remission at 6-month follow-up. However, because the study had an open-label design, it was not possible to rule out waning of OCD symptoms over time.

In response, in 2018, our group launched a randomized, double-blind, active placebo-controlled trial examining the feasibility, tolerability, safety, and clinical effects of a single moderate dose (0.25 mg/kg) of psilocybin paired with unstructured, non-directive psychological support in participants with treatment-refractory OCD (39). While this trial is still ongoing, early observations with a subsample of completers indicated clinically significant response on the Y-BOCS (i.e., < 35% reduction) at 48 h post-dosing for participants who received psilocybin (40). At the same time, there is accumulating subjective feedback post-dosing from completers in this trial about wanting a higher, fixed dose of at least 25 mg, so as to experience potentially fuller acute effects and greater benefits, especially among participants who received a lower dose of psilocybin. Completers also expressed interest in a second dose, so as to expand upon the perceived benefits from the first dose. These observations point to the need to investigate the safety and clinical effects of repeated, fixed-dose psilocybin administrations combined with non-directive psychological support for OCD in future randomized controlled designs.

Additionally, there is growing research on acute, subacute, and long-term psychological effects of psilocybin as putative mechanisms of action in alleviating various forms of psychopathology. Because psilocybin appears to have a transdiagnostic impact, the following effects/mechanisms are also of interest in the present study. Conventionally, acute psilocybin effects have been categorized as either mystical (41), insightful (42), or challenging in nature (43). Putative psychological mechanisms that may overlap with these categories can largely be subsumed under the umbrella of psychological flexibility (44), including mindfulness or cognitive defusion and radical acceptance of difficult arising internal experiences (45), as well as openness to experiences (46, 47). Closely associated mechanisms in the emotional processing arena include increased empathy (48), feelings of awe, euphoria, or other emotional breakthroughs (49), and increased spirituality (50). These psychological changes have in other words been described as constituting a “pivotal mental state” conducive to correcting dysfunctional mental processes that otherwise maintain psychopathology (51). In many ways, these mechanisms also seem complementary to the theorized “reset” mechanism of psychedelic action on the default mode network (52), albeit described at the psychological/subjective level. Additionally, while these effects and mechanisms have traditionally been assessed through self-report measures, emerging trends have converged on the utility of writing and behavioral tasks to assess changes in these constructs across time (53). In short, multimodal assessment of putative psychological mechanisms of psilocybin is an important part of understanding how and why this treatment may work for OCD. Therefore, the present study has incorporated a behavioral assessment of OCD symptoms as an exploratory measure (see Section 2.5.1.3).

In summary, we present the current study, which was developed in a data-driven manner, in response to participant feedback about wanting higher and more fixed doses of psilocybin, as well as the need for more rigorous investigations of feasibility, tolerability, safety, therapeutic effects, and potential psychological mechanisms of repeated psilocybin dosing in OCD. Specifically, this study is designed as a randomized, waitlist-controlled trial of two fixed doses of oral psilocybin paired with non-directive support, with blinded ratings. An adaptive dose selection strategy will be implemented, with the first dose being standardized at 25 mg of psilocybin, and the second dose being either the same or a higher dosage (i.e., 30 mg) (see Section 2.1 for details). The rationale for using a waitlist control condition instead of an active placebo is to mitigate challenges with premature self-unblinding among participants. Further, we chose not to go with a low dose comparator group either, due to evidence of a partial response even with low comparator doses in early psilocybin-OCD open-label research. For instance, Moreno et al. (35) expressed skepticism as to whether their lowest 25 μg/kg dose was in fact devoid of therapeutic effects. In terms of dosage selection, a study comparing weight-adjusted dosing (20 mg/70 kg and 30 mg/70 kg) and fixed dosing approaches (25 mg) for oral psilocybin among clinical samples in psilocybin-assisted therapy trials found no significant associations between subjective psychedelic effects and body weight or sex, thereby suggesting fixed-dose psilocybin oral administration as a more convenient approach (54). In this study, the possibility of escalation to 30 mg for the second dose is also responsive to participant feedback about wanting higher additional doses of psilocybin in the event of less-than-expected symptom improvement.

### 1.2 Objectives and hypotheses

**Figure 1:**
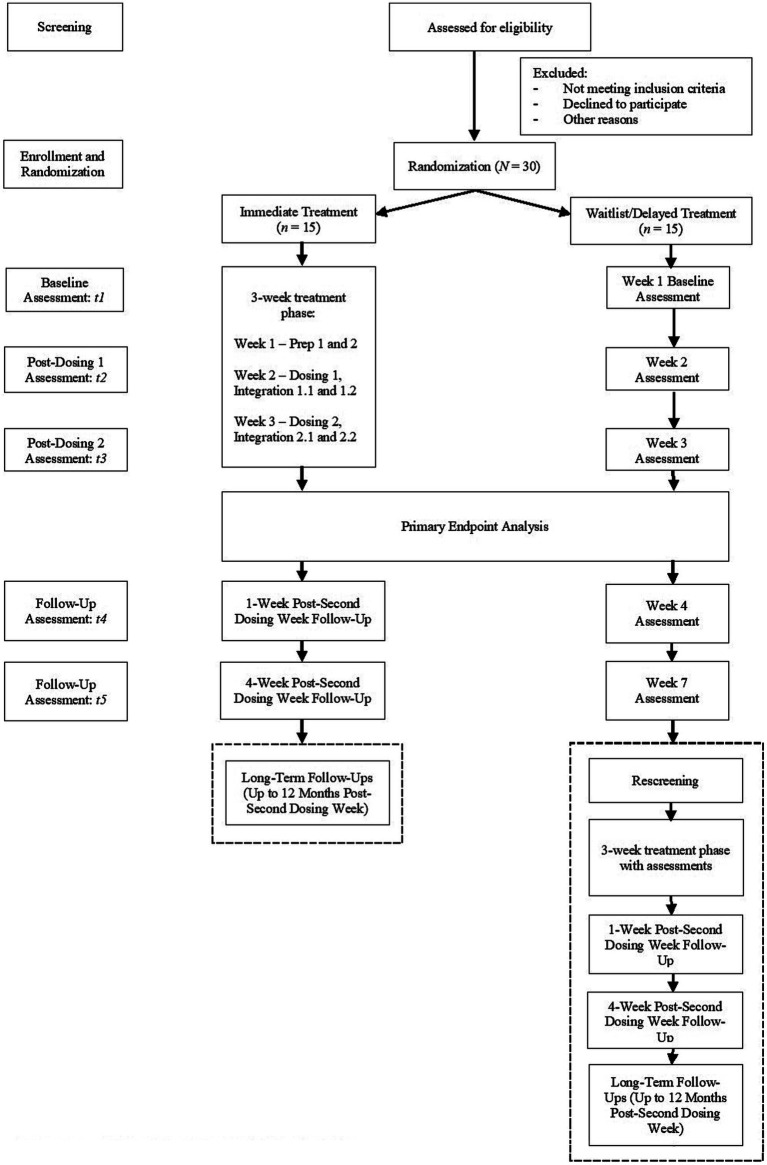
Abbreviated study flow diagram.

Other than tracking feasibility, tolerability, and safety metrics throughout the study duration (see Section 2.5.1.1), the primary objective of this study is to determine whether participants randomly assigned to immediately receive treatment (i.e., immediate treatment group) will report greater OCD symptom improvement from baseline on the Yale-Brown Obsessive-Compulsive Scale – Second Edition (Y-BOCS-II) (55) than participants randomly assigned to the waitlist/delayed treatment group. The primary endpoint is set as the end of Week 3 (see Figure 1); this corresponds to the end of the second integration session occurring 4 days post-second dose for the immediate treatment group, as well as the same interval during the waitlist phase for participants in the waitlist/delayed treatment group. We hypothesize that participants in the immediate treatment group will report statistically significantly greater mean symptom improvement from baseline on the Y-BOCS-II than participants in the waitlist group at the end of Week 3 (Hypothesis 1).

The secondary objective is to determine whether two doses of oral psilocybin will reduce OCD symptoms to a greater extent than one dose, as assessed on the Y-BOCS-II at 4 days post-each dosing. We hypothesize that the mean Y-BOCS-II score 4 days after the second dose will be significantly lower than that 4 days after the first dose (Hypothesis 2). To test this, we will aggregate treatment phase data for both groups (i.e., immediate treatment phase data for immediate treatment group, and delayed treatment phase data for waitlist group) (see Section 2.5.1.2).

The first exploratory objective of this study is to determine whether participants in the immediate treatment group will report greater improvements in other psychopathological symptoms (e.g., depressive symptoms, substance use) and OCD-related constructs (e.g., obsessive beliefs, thought suppression) from baseline than participants in the waitlist group at the same primary endpoint. The second exploratory objective is to determine whether pre-post changes (i.e., from baseline to the same primary endpoint) in putative psychological mechanisms of action will be associated with pre-post changes in OCD symptomatology.

## 2 Method

### 2.1 Trial design and setting

This trial (NCT05370911) involves a single-site, randomized (1:1), waitlist-controlled design with blinded ratings to examine the feasibility, tolerability, safety, and clinical effects of two fixed doses of oral psilocybin on OCD symptoms. Participants will be randomized to receive treatment either immediately or at a later time (i.e., delayed treatment after a 7-week waitlist phase) (see Figure 1). As with prior research (39, 56, 57), participants will receive non-directive psychological support during each dosing session, during the two preparatory sessions in the week prior to the first dosing week, as well as during the two integration sessions in the same week following each dosing (i.e., 1 day and 4 days post-dosing). All preparatory and integration sessions last for up to 2 h each. Additionally, as mentioned, an adaptive dose selection strategy will be implemented, with the first dose being standardized at 25 mg of psilocybin, and the second dose being either the same or a higher dosage (i.e., 30 mg) on the basis of a clinically significant response (≥ 35% reduction) from baseline or not, respectively, on the Y-BOCS-II at the end of the second integration session following the first dosing session (i.e., 4 days post-first dosing).^1^

This trial will be conducted from August 2023 to August 2027 at the Yale OCD Research Clinic, an outpatient clinic in the Clinical Neuroscience Research Unit (CNRU) of the Connecticut Mental Health Center (CMHC) in New Haven, CT. Duration of the study is expected to be approximately 55 and 67 weeks for participants in the immediate and waitlist/delayed treatment groups, respectively, from enrollment confirmation/randomization to the final long-term follow-up visit that occurs 12 months post-second dosing.

This study has passed Yale Human Research Protection Program (HRPP) review and has received IRB approval. The U.S. Food and Drug Administration (FDA) has granted Investigational New Drug (IND) approval (IND 134406) for this study. This study is conducted under Drug Enforcement Administration (DEA) Schedule 1 research regulations.

### 2.2 Population of interest and sample size

This study will recruit adults from 18 to 65 years old who have a primary diagnosis of OCD and a Y-BOCS-II score of at least 26, indicating at least moderately severe symptoms. The criteria for a persisting primary diagnosis of at least moderately severe OCD and the failure of at least one trial of standard care treatment (see Section 2.4.2) allow us to characterize our intended sample as treatment-refractory. We will attempt to recruit participants diversely in terms of gender, sexual orientation, and ethnoracial identity.

G*Power 3.1 was used to calculate power for a repeated-measures model with alpha of 0.05 (two-tailed), power of 0.80, and a conservative standardized effect size (*d*) of 0.5, which is considered modest in light of large effect sizes ranging beyond 1.00 in extant psilocybin studies for OCD (36) and depression (57). This analysis suggested that a total sample of 24 participants is required to yield a significant treatment group × time interaction effect at the primary endpoint, given our first objective. In budgeting for an exaggerated attrition rate of 25%, we therefore aim to enroll 30 participants in this study, which will result in an adequately powered analysis to test our primary hypothesis.

### 2.3 Study drug (psilocybin)

The study drug (psilocybin) will be manufactured, packaged, and shipped in synthetic powdered form by Usona Institute, Inc. Upon receipt, the psilocybin will be encapsulated as 25 and 30 mg capsules on-site and stored in a DEA-approved safe. Designated staff will be the only personnel with access to the psilocybin. A log of the psilocybin accountability, encapsulation, and dispensing will be kept by the designated staff. This will provide safeguarding and accounting of the investigational drug for internal and external regulatory agencies.

### 2.4 Schedule of trial activities

Figure 1 displays study flow from screening to termination, including the delayed treatment phase for participants who have been randomized to the waitlist group.

#### 2.4.1 Recruitment

The Yale OCD Research Clinic regularly sees a large number of potential participants with OCD. Participants may reach out through institutional or community referrals. Participants may also self-refer after encountering the clinic’s website, online advertisements, flyers in the community, or contact information on this study’s ClinicalTrials.gov page.

#### 2.4.2 Pre-screening, informed consent, and screening

Study personnel will follow up with potential participants who are interested in this study for phone pre-screening, informed consent, and other screening procedures. Most of these procedures/visits (e.g., phone pre-screen, informed consent visit, psychiatric evaluation) will be conducted remotely/virtually, while the remaining procedures (e.g., medical evaluation) will be conducted in-person on-site. The screening phase can take up to 4 weeks.

First, a study team member will obtain verbal consent and complete a phone pre-screen with potential participants. The purpose of this pre-screen is to gather information about participants’ demographics, personal and family psychiatric history, medical history, and substance use history. Information from this screen will be entered directly into an electronic source document. This information will be reviewed by the co-PIs to assess preliminary eligibility.

Next, preliminarily eligible participants will be scheduled to complete an informed consent visit with the co-PIs and/or appropriate study team members. This visit can occur either in-person or virtually. All study procedures will be reviewed in detail in an IRB-approved informed consent document before participants provide their signature. As part of informed consent, participants will also provide contact information for their external treating provider(s) and sign a medical release of information. This is to allow appropriate study team members to review participants’ pertinent medical records and to communicate with participants’ external provider(s) to clarify and confirm details of their treatment histories. Additionally, at this visit, participants will also designate at least one adult support person whom the co-PIs or appropriate members of the study team can correspond with in the event of an emergency, who will be expected to have continuing contact with the participant, who will provide transportation for study visits, and who may provide corroboratory feedback to the research staff about changes in participants’ behavior, mood, and attitude over the course of the study. All information gathered thus far will again be reviewed by the co-PIs to ascertain preliminary eligibility.

After obtaining informed consent, and if participants continue to be eligible, a research assistant will send survey links to more extensive self-report screening measures – demographic history form, the Diagnostic Interview for Anxiety, Mood, and OCD and Related Neuropsychiatric Disorders (DIAMOND) Self-Report Screener (58), the Structured Clinical Interview for DSM-5 Screening Personality Questionnaire (SCID-5-SPQ) (59), the Alcohol Use Disorders Identification Test (AUDIT) (60), the Drug Use Disorders Identification Test (DUDIT) (61), and the Fagerström Test for Nicotine Dependence (FTND) (62) – for participants to complete. Further, participants will also meet virtually with trained study personnel for a comprehensive psychiatric evaluation. Ahead of this visit, trained study personnel will review participants’ responses on the DIAMOND Self-Report Screener and SCID-5-SPQ to determine what modules to administer. The psychiatric evaluation will comprise a psychosocial interview and the following interviews: DIAMOND (58); Y-BOCS-II (55); Yale Global Tic Severity Scale (YGTSS) (63); Lifetime/Recent version of the Columbia-Suicide Severity Rating Scale (C-SSRS) (64); Structured Clinical Interview for DSM-5 Personality Disorders (SCID-5-PD) (59). The co-PIs will similarly review all available information at this time to ascertain preliminary eligibility.

Participants who remain eligible will go on to complete an in-person medical evaluation visit with a study physician. Procedures for this visit comprise a physical examination, blood draws, urinary drug and pregnancy screens (if of childbearing potential for the latter), liver and thyroid function tests, and an electrocardiogram (ECG). Participants will be reminded that these results will be documented on a medical chart created for them. Participants are also reminded of their right to discontinue participation at any time.

Inclusion criteria are:

1. Primary DSM-5 diagnosis of OCD on the DIAMOND
2. Y-BOCS-II score of 26 or greater at screening
3. Failure of at least one medication and/or therapy trial of standard care treatment for OCD with an adequate dose and duration, as defined by at least 12 weeks of a standard of care medication and/or at least 12 weeks of psychotherapy (CBT or ERP) for OCD^2^
4. English proficiency and fluency, and ability to understand the consent process and the risks and benefits associated with the study, and to provide informed consent
5. Willingness to sign a medical release for the study team to communicate directly with outside provider(s) to confirm medication and psychotherapy histories or arrange contingencies in event of participant crises
6. Must provide an adult contact (relative, spouse, close friend or other caregiver) who is willing and able to be reached by the co-PIs and/or study personnel in the event of an emergency, and who can provide transportation for study visits and independently comment on any changes in the participant’s mood or behavior after each administration of psilocybin.
7. Willingness to commit to all study procedures, including psilocybin dosing sessions, preparatory and integration sessions, and follow-up visits, and completing all evaluations, assessments, ratings, or measures, and commit to respond to all necessary telephone or email contacts
8. Ability to orally ingest pills for psilocybin dosing visits
9. Non-consumption of psychotropic medications [i.e., anxiolytic, neuroleptic (first- and second-generation antipsychotics), antidepressant, or mood stabilizer medications] for OCD or comorbid psychiatric conditions for at least 8 weeks at the time of randomization^3^
10. Willingness to refrain taking or starting aforementioned psychotropic medications on the days of dosing visits as well as until after 4 weeks post-second dosing week^4^
11. Willingness to cease current psychotherapy (CBT or ERP) and refrain from starting new course of psychotherapy (CBT or ERP) for OCD or comorbid psychiatric conditions until after 4 weeks post-second dosing week^5^
12. A negative urinary pregnancy screen at study entry and day of each dosing if of childbearing potential
13. Willingness to use adequate birth control and not attempt to become pregnant during study until after 4 weeks post-second dosing week

Exclusion criteria are:

1. Personal or immediate family history of formally diagnosed schizophrenia or other psychotic disorders (e.g., delusional disorder, schizoaffective disorder), or bipolar I/II disorder
2. Lack of knowledge about biological families’ medical history, due to adoption or other circumstance
3. Active suicidal intent or suicidal or non-suicidal self-injurious behaviors^6^
4. Unremitted Tourette syndrome
5. Lifetime diagnosis of autism spectrum disorder
6. Current substance use disorder (except for mild alcohol use disorder), as determined on the DIAMOND
7. Any use of classic psychedelic substances within the prior 12 months
8. Unwillingness to abstain from use of classic psychedelics outside of the study up to 4 weeks post-second dose
9. Use of tobacco products or a THC-containing product (e.g., smoked or vaporized cannabis flower, liquid or oil extract, edibles) more than 2 times per week on average over the past 30 days at screening
10. Unwillingness or inability to abstain from use of tobacco or THC-containing products from 1 week prior to randomization up to 4 weeks post-second dose
11. Positive urine drug test for any prohibited substance (i.e., amphetamines, barbiturates, buprenorphine, cocaine, methamphetamine, MDMA, methadone, opiates, and phencyclidine) at screening or days of dosing, or positive breathalyzer test for alcohol on days of dosing
12. Unwillingness or inability to abstain from alcohol use at least 24 h prior to the days of dosing, up to 24 h after each dosing day
13. Unwillingness to refrain from ingesting/using caffeine or nicotine for 2 hours before and 6 hours after ingesting the drug, or until trained research staff deem it safe to do so
14. Use of any other investigational drugs within 30 days prior to screening
15. Any neurological condition, including history of seizure(s) and/or chronic/severe headaches
16. Any history of head injury with loss of consciousness for more than 30 min
17. Hypertension at screening, defined as systolic blood pressure > 140 mmHg or diastolic blood pressure > 90 mmHg, averaged across three assessments
18. History of cardiovascular disease, including but not limited to clinically significant coronary artery disease, cardiac hypertrophy, cardiac ischemia, congestive heart failure, myocardial infarction, angina pectoris, coronary artery bypass graft or artificial heart valve, stroke, transient ischemic attack, or any clinically significant arrhythmia
19. Any clinically significant or abnormal ECG finding at screening suggestive of ischemia or infarct, complete bundle branch block, atrial fibrillation or other symptomatic arrhythmia, or predominantly non-sinus rhythm
20. Resting QT interval with Fridericia’s correction (QTcF) ≥ 450 msec (male) or ≥ 470 msec (female) at screening, or inability to determine QTcF interval
21. Presence of risk factors for torsades de pointes at screening, including long QT syndrome, uncontrolled hypokalemia or hypomagnesemia, history of cardiac failure, history of clinically significant/symptomatic bradycardia, family history of idiopathic sudden death or congenital long QT syndrome, or concomitant use of a torsadogenic medication
22. Moderate-to-severe hepatic impairment at screening, defined as a Child-Pugh score ≥ 5, alanine aminotransferase (ALT) or aspartate aminotransferase (AST) > 2 times the upper limit of normal (ULN), or bilirubin >1.5 times the ULN, unless this is attributable to Gilbert’s syndrome
23. Moderate-to-severe renal impairment at screening, defined as an estimated glomerular filtration rate of <50 mL/min/1.73 m^2^
24. Diagnosis of diabetes
25. Significant hypothyroidism or hyperthyroidism (thyroid stimulating hormone [TSH] < 0.8 times the lower limit of normal [LLN] or > 1.5 times the ULN)
26. Any other condition, disorder or finding which, in the opinion of the co-PIs, would adversely impact participant safety or the ability of the participant to complete the study, including compliance with all study requirements and procedures

#### 2.4.3 Enrollment confirmation and randomization

After a participant has been screened to be eligible, their enrollment will be confirmed by the co-PIs via a confirmation email and/or phone call. At this time, participants will also be randomized by the co-PIs to either the immediate treatment or waitlist/delayed treatment groups and begin study visits. One of the co-PIs will generate the block randomization sequence (i.e., equal number of participants in each treatment condition) with an online program (randomizer.org).

#### 2.4.4 Blinding

Given the choice of a waitlist control condition, only the independent raters will be blinded to participants’ condition assignment in this study. During the blinded ratings phase (7 weeks from randomization) for either condition, participants will be required to not disclose their assigned treatment condition to the independent raters. Additional measures to maintain blinding of independent raters include having all rating visits during the blinded phase be conducted remotely/virtually, and for all participants to turn their video off (on Zoom) during these visits so as to not reveal their background location (i.e., at the study site vs. the participant’s home). Should the blind need to be broken in the event of an adverse event or other emergency (e.g., active suicidal risk detected on the C-SSRS by the independent rater during follow-up visits), said emergency will be reported to the co-PIs, who will then disclose the assignment condition as necessary to the independent raters.

#### 2.4.5 Study visits with facilitators

Over the course of several study visits, participants meet and work with their assigned pair of facilitators in preparatory, dosing, and integration sessions. Each participant will work with their assigned pair of facilitators for all sessions.

Essential procedures for preparatory, dosing, and integration sessions in the treatment phase – which are identical for both the immediate and waitlist conditions – are described in Sections 2.4.5.2 to 2.4.5.4. Detailed guidelines for all study facilitators’ activities are described in a separate facilitator manual (65). The appendices in said manual also contain flexible, easy-to-use checklists for facilitators for each type of visit.

##### 2.4.5.1 Non-directive approach

In all sessions, facilitators provide psychological support using a non-directive approach, which we have operationalized as a participant-centered/−led process, in which facilitators are attentive, empathic, and welcoming of each participant’s unfolding and varied experiences prior to, during, and after each dosing. Through a non-directive approach, facilitators allow time for participants to reach their own conclusions and insights, through the use of silence, reflective listening, or summary statements. This approach also allows for the use of more directive or instructive communication by facilitators under certain circumstances (e.g., intervening with grounding strategies to redirect from acute distress during dosing), to remain responsive to the participant’s evolving needs.

In adopting a non-directive approach to psychological support, facilitators attune to psychological processes common in OCD that may influence the nature or trajectory of preparatory, dosing, and integration sessions. These include, but are not limited to: perfectionism; intolerance of uncertainty; inflated responsibility and overestimation of threat; importance of and need to control thoughts; low distress tolerance; intolerance of uncontrollability; experiential avoidance; self-criticalness; mindlessness; and psychological inflexibility/rigidity (66–70). We have described this approach, and the aforementioned psychological processes in OCD as they may apply to study visits, at length in the same facilitator manual mentioned above (65).

##### 2.4.5.2 Preparatory sessions

During the two in-person preparatory sessions, participants will work with their assigned pair of study facilitators, and begin building rapport and trust, as well as cultivate a safe and comfortable environment for the upcoming dosing sessions. To do this, facilitators collaborate with participants to learn about: how participants live with OCD and the impact of OCD on their quality of life; partiicpants’ values and beliefs; their psychosocial and treatment histories, including significant past experiences/trauma history; any prior experience(s) with psychedelics or altered states of consciousness; and their specific intentions/goals and expectations/assumptions for the upcoming dosing sessions.

In these sessions, facilitators also provide psychoeducation on psilocybin’s acute effects, the facilitators’ non-directive approach during dosing, and ways for participants to ground themselves from challenging experiences during dosing (e.g., maintaining openness and curiosity; removing headphones or eye shades as a temporary way to ‘drop out’ of the experience; deep breathing; reminding participants that drug effects, while challenging, are transient). Further, facilitators collaborate with participants to reach agreements about maintaining safety and consensual boundaries around common-sense, supportive, non-sexual touch during dosing. Examples of supportive touch may involve holding of hands, or offering a hand on the participant’s shoulder.

Additionally, during these sessions, facilitators remind participants of the need to adhere to relevant lifestyle modifications (e.g., limit or refrain from exclusionary substances per protocol, ingesting a light, low-fat breakfast the morning of each dosing day), and discuss the importance of adequate rest and a lighter schedule sleep prior to and after each dosing day. Lastly, facilitators will answer any of participants’ remaining questions and obtain input on how to adjust aspects of the physical setting (e.g., location of furniture, type of hanging art) so as to promote comfort during dosing. Throughout these preparatory sessions, facilitators are alert to any new or unforeseen factors that may be contraindicatory to dosing. Through these sessions, participants presumably reach a psychological state (i.e., set) (71) conducive to the first dosing session.

##### 2.4.5.3 Dosing sessions

Participants arrive on-site during the early morning of each dosing day to complete pre-dosing screens for drug, alcohol, and pregnancy (if of childbearing potential) and other assessments (e.g., about medication changes, self-reports) with a research assistant and a study physician. By approximately 10 am, participants will settle into the dosing room with the study facilitators to begin the dosing session. The study pharmacy coordinator will retrieve and deliver the study drug by 10 am, remaining in the room until the participant has ingested the study drug. One facilitator will monitor the participant’s vitals (blood pressure, heart rate, temperature) and subjective units of distress (SUDS; from 0 to 100) at baseline prior to ingestion of the study drug, as well as at subsequent intervals until the end of each dosing session (i.e., 15 min and 30 min post-dosing, then every 30 min after until 2 h post-dosing, and finally every hour after until end of dosing session).

After the psilocybin is ingested, facilitators will encourage participants to assume a comfortable position on the bed with the headphones and eye shades on, and to listen to a standardized music playlist. This playlist is curated to facilitate the prototypical arc of a psilocybin experience. In our experience, this arc is as follows: ‘ascent’/onset of effects (0–1.5 h), ‘peak’ effects in one or more ‘waves’ (1.5 to 3.5 h), ‘descent’/subsiding of effects (3.5 to 5 h), and ‘return’ to usual state (5 h and beyond). The use of eye shades and headphones with music helps eliminate extraneous distractions and provides an immersive way to “drop into” the dosing experience. Participants may ask to pause the music, adjust the volume, or briefly remove the headphones and eye shades to take a bathroom break, snack and hydrate, or to receive support for arising experiences from the facilitators.

Throughout each dosing session, facilitators will attend to participants’ physical and psychological needs, while ensuring their safety (65). At least one facilitator will be physically present in the room with the participant at all times. Facilitators will be attentive to participants’ reactions to any arising internal experiences. Facilitators also support participants in openly navigating these experiences in a non-directive, compassionate, and empathetic manner. As mentioned, if needed, facilitators will implement grounding strategies and/or offer supportive, consensual touch to reduce participants’ distress and empower them to approach and ‘move through’ challenging experiences. If participants are not responsive to these strategies after repeated attempts and/or if participants are at acute risk of harming themselves or others, one of the co-PIs or the study physician will prescribe and administer a rescue medication (benzodiazepine), stepping up interventions as needed.

For each dosing session, facilitators will stay with, observe, and support participants until there is significant dissipation of psilocybin effects, which typically occurs around 5–6 h after ingestion. For some participants though, this may take up to 8 h. At the end of the session, facilitators will inquire about AEs and whether participants are still experiencing any residual drug effects. If participants report any persisting drug effects, acute suicidality, or other SAEs, they will continue to be monitored on the inpatient unit. The co-PIs/study physician will implement further interventions and procedures (e.g., transfer to nearest emergency department, longer-term psychiatric hold on the unit, or withdrawal and referrals for treatment) as necessary. Otherwise, the study physician will assess and medically clear participants to leave the study site as accompanied by their support person. Prior to participants leaving the study site, facilitators will encourage participants to reserve further discussion of their dosing experience for the integration visits, instead of processing their experiences with people outside of the study team while still in the treatment phase.

##### 2.4.5.4 Integration sessions

After each dosing session, facilitators will continue to work with participants in two integration sessions within the same week, for a total of four integration visits during each participant’s treatment phase. Both facilitators will be in attendance at all integration sessions. While these integration sessions can be in-person, participants have the option of attending these visits virtually if there are no same-day, in-person study procedures.

During integration sessions, facilitators will support participants in narrating and processing their dosing experiences (65). Facilitators assume the same attentive, non-directive, participant-led approach to these discussions. These integration sessions presumably offer participants a supportive setting to (re)consolidate any emergent insights during and after each dosing session. As such, participants may arrive at new ideas or conclusions about how to, for example, manage and live with OCD moving forward. This interaction or synergy between psilocybin and facilitator effects and participants’ processing throughout the treatment phase has been described elsewhere (72). During these sessions, facilitators will also document AEs (e.g., new or persisting drug effects), and intervene if necessary.

#### 2.4.6 Waitlist phase

Participants in the waitlist group do not meet with facilitators until raters are unblinded (see Section 2.4.7) and waitlist participants are rescreened successfully and have started their delayed treatment phase (see Section 2.4.8). During the waitlist phase, participants meet with an independent rater at the same intervals as participants in the immediate treatment group.

#### 2.4.7 Unblinding

Unblinding of independent raters will occur immediately after the ratings have been obtained at the 4-week post-second dosing interval for participants in the immediate treatment group, which is the same as the 7-week post-randomization interval for participants in the waitlist group.

#### 2.4.8 Rescreening and delayed treatment phase

Participants randomly assigned to the waitlist group will be medically rescreened upon breaking of independent raters’ blind to ensure that they still are eligible to participate in the study prior to starting their delayed treatment phase. Rescreening procedures will consist of the same medical evaluation procedures described in Section 2.4.2. All procedures for preparatory, dosing, and integration sessions during the delayed treatment phase for the waitlist group are identical to those for the immediate treatment group, except that all independent rater evaluations are unblinded. Waitlist participants will be re-assessed on all study measures based on the same schedule as for participants in the immediate treatment group. For descriptive purposes, we will collect data from waitlist participants during their delayed treatment phase. However, these data will only be included in secondary analyses of psilocybin’s effects on OCD and related symptomatology.

#### 2.4.9 Follow-ups

For all participants, follow-up visits will occur 1 and 4 weeks after the end of the second dosing week. Long-term follow-up for all participants will occur at 3, 6, and 12 months after the end of the second dosing week. In these follow-up visits, participants complete self-reports and evaluations by independent raters.

#### 2.4.10 Optional trial component

##### 2.4.10.1 Qualitative interview

An independent interviewer will conduct a qualitative interview with participants approximately 1 month after the end of the second dosing week. Since this is an optional component of the trial, participants will be required to provide additional consent to participate, as well as for the interview to be recorded for the purposes of transcription and subsequent qualitative analysis. Consenting for the qualitative interview occurs for all participants at the informed consent visit (Section 2.4.2). The goal of this interview is to obtain qualitative data about participants’ pre-study symptoms and functioning, their psilocybin dosing experiences (e.g., acute perceptual experiences, persisting insights or realizations), as well as potential changes in OCD symptoms, functioning, and quality of life approximately 1 month after the end of the second dosing week.

### 2.5 Outcomes

#### 2.5.1 Clinical outcomes

##### 2.5.1.1 Primary outcomes

The primary outcome measure is the Y-BOCS-II (55), which assesses OCD symptom severity over the past week. This will be administered by an independent rater at baseline prior to the first preparatory session, at the end of each dosing week (i.e., after the second integration session for each week), and at the follow-up intervals of 1 and 4 weeks and 3, 6, and 12 months after the end of the second dosing week.

We also include safety outcomes as primary outcomes. Safety measures completed during dosing include vitals and SUDs. Safety measures completed throughout the study duration include the Since Last Visit version of the C-SSRS (64), AEs, and any medication changes. The Since Last Visit version of the C-SSRS (64) assesses suicidal ideation severity, ideation intensity, and suicidal behavior severity (including non-suicidal, self-injurious behaviors) since the last assessment interval. AEs are defined as any untoward physical, social, economic, or psychological occurrence affecting participants, including any abnormal laboratory finding, symptom, reaction, or disease. The CTCAE v5.0 will be used as a classification system to categorize and label AEs and to grade their severity (from 1 to 5) in this trial (73). It is not necessary for an AE to have a causal relationship with study procedures. The severity of an AE is also distinct from its ‘seriousness.’ An AE is considered serious (i.e., SAE) if it: (1) is life-threatening; (2) results in hospitalization, disability/incapacity, a congenital anomaly or birth defect, or death; (3) requires medical or surgical intervention to prevent aforementioned outcomes; or (4) adversely affects the risk/benefit ratio of the study. As such, a severe AE is may not always count as an SAE.

We evaluate feasibility and tolerability based on the following metrics. First, we will report the total number of enrolled, screened, eligible, enrollment-confirmed, and randomized participants. We will also report on the total number of participants who were ineligible, who voluntarily withdrew from participation prior to or after randomization, and who were withdrawn by the co-PIs due to safety concerns. To evaluate the restrictiveness of our eligibility criteria, we will report the number of participants excluded based on different criteria. Additionally, we will report follow-up rates (i.e., number who completed follow-up divided by the number of randomized participants) by treatment arm for all study visits up to 12 months after the end of the second dosing week. Number and percentage withdrawal from the trial with reasons for withdrawal will be summarized at each follow-up and by treatment arm. We will also summarize the number and percentage of participants with missing data for primary and secondary outcomes as a whole and by treatment group and follow-up intervals up to 12 weeks post-dosing. If possible, we will conduct logistic regressions to assess baseline characteristics of completers against those of participants with missing data at each follow-up interval.

##### 2.5.1.2 Secondary outcome

The secondary outcome in this study is also OCD symptom severity measured by the Y-BOCS-II; the difference is that we will focus on and aggregate Y-BOCS-II scores obtained from the treatment phase across groups. In other words, we will combine Y-BOCS-II data collected during the immediate treatment phase for the immediate treatment group with Y-BOCS-II data collected during the delayed treatment phase for the waitlist group for the secondary outcome.

##### 2.5.1.3 Exploratory outcomes

Exploratory outcomes are assessed by various rater−/evaluator-administered or self-report measures. These measures will be administered at baseline, immediately before or after each dosing, and/or at varying follow-up intervals up to 12 months after the end of the second dosing week. These measures include:

1. Montgomery-Åsberg Depression Scale (MADRS) (74)
2. Dimensional Obsessive-Compulsive Scale (DOCS) (75)
3. Obsessive Beliefs Questionnaire-44 (OBQ-44) (70)
4. Acceptance and Action Questionnaire for Obsessions and Compulsions (AAQ-OC) (76)
5. Tolerance of Uncontrollability Questionnaire (TOUQ) (66)
6. White Bear Suppression Inventory (WBSI) (77)
7. Difficulties in Emotion Regulation Scale (DERS) (78)
8. Southampton Mindfulness Questionnaire (SMQ) (79)
9. Toronto Mindfulness Scale (TMS) (80)
10. Set, Setting, and Intentions (SSI) (81)
11. Mystical Experience Questionnaire (MEQ) (41)
12. Psychological Insight Questionnaire (PIQ) (42)
13. Challenging Experience Questionnaire (CEQ) (43)
14. Ego Dissolution Inventory (EDI) (82)
15. Emotional Breakthrough Inventory (EBI) (83)
16. Persisting Effects Questionnaire (PEQ) (50)
17. Self-Compassion Scale (SCS) (84)
18. Ten-Item Personality Inventory (TIPI) (85)
19. Alcohol Use Disorders Identification Test (AUDIT) (60)
20. Drug Use Disorders Identification Test (DUDIT) (61)
21. Fagerstrom Test for Nicotine Dependence (FTND) (62)
22. Quality of Life Enjoyment & Satisfaction Questionnaire-Short Form (Q-LESQ-SF) (86)
23. World Health Organization Disability Assessment Schedule Version 2.0 (WHODAS-2.0) (87)
24. Working Alliance Inventory-Short Revised (WAI-SR) (88)
25. Stanford Expectations of Treatment Scale (SETS) (89)
26. Modified version of the Theoretical Orientation Profile Scale-Revised (TOPS-R) (90); completed by facilitators only

Additionally, participants will complete an OCD symptom provocation task (SPT) adapted from Tendler et al.’s (91) protocol at baseline and at the end of the second dosing week. In this task, the evaluator will collaborate with participants to formulate idiosyncratic, moderately distressing (SUDS of 4–7 on scale of 0 [not distressing at all] to 10 [worst level of distress ever]), and uncertainty-provoking questions anchored to their primary obsessions. When the task commences, participants will write down these questions on a blank piece of paper with a pen, and mentally repeat those questions for 5 min without engaging in any compulsions. Immediately afterwards, participants will complete manipulation check items assessing effort and vividness of thoughts and images elicited during the symptom provocation phase. If these effort or vividness ratings fall below 5 [on a scale of 0 (no effort at all/not vivid at all) to 10 (most effort ever/most vivid ever)], participants will be required to repeat another 5 min of symptom provocation with additional instructions to invest more effort into mentally repeating these questions. Participants will then rate their distress (on the same 0–10 scale) at the start, middle, and end of the (most recent) symptom provocation phase. Thereafter, participants enter a free-compulsion phase that lasts 15 min, where they have the option of physically neutralizing their distress and uncertainty in various ways, such as tearing up the piece of paper, crossing out or rephrasing the questions, or writing counter statements. During this phase, participants will also be permitted to engage in any overt and covert compulsions. Participants will be monitored by the evaluator, who will document behavioral observations. At the end of this phase, participants complete: 0–10 ratings of distress and compulsive urges at the start, middle, and end of the free-compulsion phase; whether compulsions were performed (yes/no), regardless of whether they were overt or covert; description of each compulsion, and whether these were overt or covert; number of times each compulsion was performed; and duration of each compulsion in minutes and seconds.

Lastly, participants will complete a writing task at baseline and at the end of the second dosing week. In this task, participants describe their perceptions of their OCD symptoms based on the following prompt: “For the next 15 min, please write down in detail your thoughts and feelings about your OCD symptoms at this point in time. There are no right or wrong responses. Write down the first things that come to your mind. Please write as much as possible. There is no limit to how much you can write.” The purpose of this task is to generate data for exploratory mixed-methods analysis of psycholinguistic predictors, mechanisms, or moderators of treatment response.

#### 2.5.2 Evaluation of success of blinding

Immediately prior to unblinding of the independent rater at the end of 7 weeks post-randomization for each participant, the independent rater will be verbally probed to name the treatment arm they believe each participant was assigned to, with this response documented on the corresponding visit note.

### 2.6 Facilitator competencies

A separate manual (65) describes: required credentials, expected core competencies, and roles and responsibilities of facilitators; recommended facilitator dyad dynamics; as well as guidelines for facilitating preparatory, dosing, and integration sessions per a non-directive approach. Treatment fidelity is not assessed in this trial because we will not be testing a structured program of psychological support. Rather, facilitators are expected to: attest that they have reviewed and understood the facilitator manual prior to working with their first participant; engage in peer debrief and supervision with their co-facilitator; and regularly attend weekly rounds to process and discuss their interactions with participants with the rest of the study team. These measures ensure that facilitators are facilitating sessions according to guidelines set out in the manual.

### 2.7 End of trial

End of trial refers to the interval at which the database is locked from data entry, which may coincide with or be farther out from the last time point of data collection (i.e., 12-month post-second dosing follow-up interval with the final completer, whichever condition they are in).

### 2.8 Trial monitoring

The co-PIs will convene a Data and Safety Monitoring Board (DSMB) comprising: (1) a psychologist; (2) a psychiatrist with expertise in acute pharmacological effects; and (3) a psychiatrist with clinical trial expertise. The co-PIs and DSMB will ensure safety oversight by monitoring the data, assuring protocol compliance, and conducting safety reviews. The first safety review will be conducted after 5 participants have completed the 4-week post-second dosing follow-up visit. Subsequent safety reviews will be conducted every 6 months after that, including when reapproval of the protocol is sought. During the review process, the co-PIs will collaborate with the DSMB to evaluate whether the study should continue unchanged, require modification or amendment, or close to enrollment. The co-PIs will communicate the latter two decisions as soon as possible to the IRB/Human Investigations Committee (HIC). The research team will also work with the monitoring team at the Yale Center for Clinical Investigation (YCCI) to conduct regular clinical monitoring procedures, including accurate grading of AEs and following up on SAEs, as well as necessary follow-up IND reporting.

The co-PIs, DSMB, CMHC, IRB/HIC, or the FDA will have the authority to stop or suspend the study, or require modifications. If the trial is prematurely stopped or terminated (e.g., due to an unacceptable tolerability profile, unacceptably high rate of protocol deviations), the co-PIs will be required to promptly inform active study participants, and provide appropriate therapy referrals if necessary and follow-up. All procedures and requirements pertaining to retention and storage of documents (see Section 2.9) will still be observed. All other study materials will be treated in accordance with federal and state regulations.

### 2.9 Data collection, storage, and security

We will conduct this study within organizations fully bound by and compliant with HIPAA policies and strict research requirements. We will record data in written and/or electronic formats. Paper source documents/charts will be stored in locked filing cabinets in a locked office. Electronic records and data will be stored as password-protected files on secured computer(s), on secure server(s), and/or on a secure data capture system. Access to any data will be restricted to the co-PIs and relevant study team members with appropriate training. Personal identifiers and protected health information (PHI) can be accessed only by the co-PIs and approved research staff. Only deidentified data will be used for data analysis and dissemination efforts (e.g., presentations, publications) resulting from this study. We have obtained a Certificate of Confidentiality (CoC) from the FDA for this study, which provides further protection for participant data confidentiality. Study documents will be retained indefinitely. The co-PIs in collaboration with the YCCI monitoring team will periodically review data collection, storage, and distribution processes and practices, and implement changes to enhance confidentiality and privacy if necessary.

### 2.10 Statistical analysis

We will conduct linear mixed modeling (LMM) and correlational analyses using SPSS and/or R software to test our primary and secondary hypotheses, as well as to examine our exploratory objectives. For LMMs, restricted maximum likelihood estimation will be used to handle missing data and maximize the inclusion of all randomized participants. Treatment group (immediate treatment vs. waitlist/delayed treatment), time, and all interactions will be modeled as fixed effects. Subjects will be modeled as a random effect.

Analyses will be based on modified intent-to-treat (ITT) sets. For Hypothesis 1 and the first exploratory objective, the ITT set will comprise participants who completed at least the first post-dosing assessment or the equivalent interval during the blinded ratings phase. For Hypothesis 2 and the second exploratory objective, the ITT set comprises participants who completed at least the first post-dosing assessment during the respective treatment phases.

To test Hypothesis 1, we will focus on Y-BOCS-II scores at the primary endpoint of 4 days post-second dose (or the equivalent interval for waitlist participants). The significance of the treatment group × time (baseline vs. 4 days post-second dose) interaction effect at alpha = 0.05 (two-tailed) is of interest, with the second psilocybin dosage (25 vs. 30 mg) for the immediate treatment group included as a covariate. Alpha-corrected t-tests will be used to probe a significant interaction effect.

To test Hypothesis 2, we will focus on Y-BOCS-II scores 4 days post-first dose and 4 days post-second dose during the respective treatment phases. The significance of the time (4 days post-first dose vs. 4 days post-second dose) effect at alpha = 0.05 (two-tailed) is of interest. Covariates include baseline Y-BOCS-II scores, the second psilocybin dosage (25 or 30 mg), and whether ratings were blinded.

To examine the first exploratory objective, we will focus on scores on measures of relevant exploratory outcomes (see Section 2.5.1.3) at the same primary endpoint of 4 days post-second dose (or the equivalent interval for waitlist participants). Similarly, the significance of the treatment group × time (baseline vs. 4 days post-second dose) interaction effect at alpha = 0.05 (two-tailed) is of interest here. The second psilocybin dosage (25 vs. 30 mg) for the immediate treatment group will be included as a covariate. Alpha-corrected t-tests will be used to probe a significant interaction effect.

To examine the second exploratory objective, we will focus on pre-post changes (baseline vs. 4 days post-second dose) in Y-BOCS-II scores and scores on measures of putative psychological mechanisms of action (OBQ-44, AAQ-OC, etc.). Part correlations will be run to test whether these change scores are significantly correlated, after controlling for the second psilocybin dosage (25 or 30 mg) and whether ratings were blinded.

Safety data will be presented as descriptive statistics, followed up with appropriate parametric (e.g., independent t-tests) and non-parametric analyses (e.g., chi-square tests of independence) to compare safety data between treatment groups at various intervals. Data from the one-month post-dosing interviews will be qualitatively analyzed via interpretative phenomenological analysis (IPA) (92).

### 2.11 Ethics and dissemination

The Yale University HIC has approved this trial (#2000032623). This trial was also registered with ClinicalTrials.gov (NCT05370911). The Yale University HIC will review and approve protocol modifications prior to the co-PIs updating relevant information on ClinicalTrials.gov. We will seek reconsent from participants, if necessary. We will also submit a manuscript describing the primary outcome for publication in a peer-reviewed journal, prior to submitting manuscripts for secondary outcomes and qualitative data, as appropriate. Additionally, the results from this trial may be publicized via other media (e.g., posters, presentations, interviews).

## 3 Discussion

This is the first-ever published study protocol for a randomized, two-dose, waitlist-controlled, rater-blinded trial of psilocybin combined with non-directive psychological support for OCD. If repeated psilocybin administration is well-tolerated, feasible, safe, and leads to a fast and significant reduction in OCD symptoms, this study would mark a new frontier in treatment options for refractory OCD. With exploratory analyses of putative psychological mechanisms underlying the effects of psilocybin with non-directive support on OCD, the present study may also catalyze future research seeking to optimize the process of psilocybin treatment for OCD.

This study has a few strengths, such as the use of a randomized design with long-term follow-ups and blinded ratings on an updated and psychometrically robust version of a gold-standard primary outcome measure (i.e., the Y-BOCS-II). The present study was also designed in response to converging qualitative feedback about the viability of higher and more psilocybin doses from completers in a current single-dose trial. Additionally, the dose escalation strategy focuses on whether participants showed a clinically significant response after the first dose, instead of whether the first dose was well-tolerated, an arguably more subjective threshold commonly used in previous psilocybin research. Moreover, the choice of a waitlist/delayed treatment group helps control for regression in OCD symptom severity over time. The waitlist-control design also acknowledges the synergistic interactions among psilocybin effects, facilitator effects, and other set or setting factors in contributing to clinical effects, contrary to placebo control designs, which purport to isolate psilocybin effects despite clear blinding issues. Further, the type of psychological support has also been transparently detailed in a separate manual (65), and facilitator activities will be assessed with a modified version of the TOPS-R, which may provide more information about extra-pharmacological moderators of treatment response.

There are also a few limitations to this study. A waitlist/delayed treatment group has its own share of expectancy effects (e.g., feelings of well-being due to the prospect of later treatment). It remains unknown whether organic preparations of psilocybin (i.e., fresh or dried psilocybin-containing mushrooms that also contain baeocystin) would show the same proposed effects as the synthetic psilocybin used in this trial. There is also the possibility of a selection bias for the qualitative interview, in that participants who have benefitted from the study may be more likely to consent to the interview than participants who either experienced negative reactions to psilocybin dosing or did not experience a clinically significant response to treatment. This is also a single-site study with a small, highly screened sample of participants who, although are treatment-refractory, do not present with acute risk. Our sample is also intended to be relatively medically healthy owing to the dose ranges used in this study. These characteristics likely limit generalizability of eventual findings to diverse, low-severity, medically fragile populations with OCD.

## Ethics statement

The trial was approved by the Yale University HIC (#2000032623). All participants will be required to provide written informed consent.

## Author contributions

TC: Conceptualization, Investigation, Methodology, Project administration, Writing – original draft, Writing – review & editing. LA: Project administration, Writing – review & editing. CB: Project administration, Writing – review & editing. ED’A: Project administration, Writing – review & editing. JE: Project administration, Writing – review & editing. TE: Project administration, Writing – review & editing. MF: Project administration, Writing – review & editing. GF: Project administration, Writing – review & editing. RG: Investigation, Project administration, Writing – review & editing. JH: Investigation, Project administration, Writing – review & editing. AJ: Project administration, Writing – review & editing. SK: Project administration, Writing – review & editing. BM: Investigation, Project administration, Writing – review & editing. PP: Project administration, Writing – review & editing. HS: Project administration, Writing – review & editing. SS: Project administration, Writing – review & editing. CW: Project administration, Writing – review & editing. CP: Funding acquisition, Investigation, Resources, Writing – review & editing. BK: Funding acquisition, Investigation, Resources, Writing – review & editing.

## Footnotes

^1^In the event that dosing-related SAE(s) occur during the first dosing session in the absence of a clinically significant response, and the participant continues in the study, the co-principal investigators (co-PIs) will consider retaining the second dose at 25 mg for safety, instead of escalating to 30 mg.

^2^To meet this requirement, participants must provide clinical documentation of past treatment(s) for OCD from their outside treating provider(s), which will be assessed against this criterion.

^3^Participants will work with their prescriber(s) to complete any medication tapering. The study team would not be managing this process.

^4^The exceptions are: benzodiazepine PRN use up to 3 days before each dosing session, as well as a stable dose of a benzodiazepine for sleep or a non-benzodiazepine sleeping agent for at least 6 weeks – participants on this stable dose will be allowed to continue these medications on this fixed dose throughout the study period; prescribed opiate pain medications or over-the-counter non-narcotic pain medications, which can be taken as prescribed or recommended at any time. The co-PIs may also prescribe a one-time dose of benzodiazepine as a rescue medication for psychological distress as necessary during dosing sessions, or prescribe a one-time dose of zolpidem for insomnia as needed during the study period. Should participants’ external providers recommend starting a new psychiatric medication, or if participant desires, participant will be required to notify the study team.

^5^Should participants’ external providers recommend restarting psychotherapy, or if participant desires, participant will be required to notify the study team.

^6^Active suicidal intent is defined by a “yes” response to either questions 4 or 5 on the Lifetime/Recent version of the C-SSRS within the past 3 months at screening, or a “yes” response to either questions 4 or 5 on the Since Last Visit version of the C-SSRS prior to each dosing. Active suicidal or non-suicidal self-injurious behaviors are defined by “yes” responses to the relevant behavior items on the Lifetime/Recent version of the C-SSRS within the past 3 months at screening, or on the Since Last Visit version of the C-SSRS prior to each dosing.

## References

[1] Kessler RC Petukhova M Sampson NA Zaslavsky AM Wittchen HU. Twelve-month and lifetime prevalence and lifetime morbid risk of anxiety and mood disorders in the United States. Int J Methods Psychiatr Res. (2012) 21:169–84. doi: 10.1002/mpr.1359, PMID: 22865617 PMC4005415

[2] Macy AS Theo JN Kaufmann SC Ghazzaoui RB Pawlowski PA Fakhry HI . Quality of life in obsessive compulsive disorder. CNS Spectr. (2013) 18:21–33. doi: 10.1017/S1092852912000697 23279901

[3] Ruscio AM Stein DJ Chiu WT Kessler RC. The epidemiology of obsessive-compulsive disorder in the National Comorbidity Survey Replication. Mol Psychiatry. (2010) 15:53–63. doi: 10.1038/mp.2008.94 18725912 PMC2797569

[4] Subramaniam M Soh P Vaingankar JA Picco L Chong SA. Quality of life in obsessive-compulsive disorder: impact of the disorder and of treatment. CNS Drugs. (2013) 27:367–83. doi: 10.1007/s40263-013-0056-z 23580175

[5] da Conceição Costa DL de Campos AP de Bragança Pereira CA Torres AR Dos Santos AC Requena G . Latency to treatment seeking in patients with obsessive-compulsive disorder: results from a large multicenter clinical sample. Psychiatry Res. (2022) 312:114567. doi: 10.1016/j.psychres.2022.114567, PMID: 35490573

[6] Soomro GM Altman DG Rajagopal S Oakley Browne M Cochrane Common Mental Disorders Group. Selective serotonin re-uptake inhibitors (SSRIs) versus placebo for obsessive compulsive disorder (OCD). Cochrane Database Syst Rev. (2008) 2008:CD001765. doi: 10.1002/14651858.CD001765.pub3, PMID: 18253995 PMC7025764

[7] Carpenter JK Andrews LA Witcraft SM Powers MB Smits JAJ Hofmann SG. Cognitive behavioral therapy for anxiety and related disorders: a meta-analysis of randomized placebo-controlled trials. Depress Anxiety. (2018) 35:502–14. doi: 10.1002/da.22728, PMID: 29451967 PMC5992015

[8] Cuijpers P Sijbrandij M Koole SL Andersson G Beekman AT Reynolds CF III. The efficacy of psychotherapy and pharmacotherapy in treating depressive and anxiety disorders: a meta-analysis of direct comparisons. World Psychiatry. (2013) 12:137–48. doi: 10.1002/wps.20038, PMID: 23737423 PMC3683266

[9] Öst LG Havnen A Hansen B Kvale G. Cognitive behavioral treatments of obsessive-compulsive disorder. A systematic review and meta-analysis of studies published 1993-2014. Clin Psychol Rev. (2015) 40:156–69. doi: 10.1016/j.cpr.2015.06.003, PMID: 26117062

[10] Reid JE Laws KR Drummond L Vismara M Grancini B Mpavaenda D . Cognitive behavioural therapy with exposure and response prevention in the treatment of obsessive-compulsive disorder: a systematic review and meta-analysis of randomised controlled trials. Compr Psychiatry. (2021) 106:152223. doi: 10.1016/j.comppsych.2021.152223, PMID: 33618297

[11] Issari Y Jakubovski E Bartley CA Pittenger C Bloch MH. Early onset of response with selective serotonin reuptake inhibitors in obsessive-compulsive disorder: a meta-analysis. J Clin Psychiatry. (2016) 77:e605–11. doi: 10.4088/JCP.14r09758, PMID: 27249090

[12] Fineberg NA Reghunandanan S Brown A Pampaloni I. Pharmacotherapy of obsessive-compulsive disorder: evidence-based treatment and beyond. Aust N Z J Psychiatry. (2013) 47:121–41. doi: 10.1177/0004867412461958, PMID: 23125399

[13] Pallanti S Hollander E Bienstock C Koran L Leckman J Marazziti D . Treatment non-response in OCD: methodological issues and operational definitions. Int J Neuropsychopharmacol. (2002) 5:181–91. doi: 10.1017/S1461145702002900 12135542

[14] Stein DJ Koen N Fineberg N Fontenelle LF Matsunaga H Osser D . A 2012 evidence-based algorithm for the pharmacotherapy for obsessive-compulsive disorder. Curr Psychiatry Rep. (2012) 14:211–9. doi: 10.1007/s11920-012-0268-9, PMID: 22527872

[15] Bloch MH Green C Kichuk SA Dombrowski PA Wasylink S Billingslea E . Long-term outcome in adults with obsessive-compulsive disorder. Depress Anxiety. (2013) 30:716–22. doi: 10.1002/da.22103, PMID: 23532944 PMC3932438

[16] Grant JE Mancebo MC Weinhandl E Odlaug BL Eisen JL Rasmussen SA. Longitudinal course of pharmacotherapy in obsessive-compulsive disorder. Int Clin Psychopharmacol. (2013) 28:200–5. doi: 10.1097/YIC.0b013e3283613e4d, PMID: 23587985 PMC3920831

[17] Levy HC O'Bryan EM Tolin DF. A meta-analysis of relapse rates in cognitive-behavioral therapy for anxiety disorders. J Anxiety Disord. (2021) 81:102407. doi: 10.1016/j.janxdis.2021.102407, PMID: 33915506

[18] Keleher J Jassi A Krebs G. Clinician-reported barriers to using exposure with response prevention in the treatment of paediatric obsessive-compulsive disorder. J Obsessive Compuls Relat Disord. (2020) 24:100498. doi: 10.1016/j.jocrd.2019.100498, PMID: 32140386 PMC7043329

[19] Koran LM Hanna GL Hollander E Nestadt G Simpson HB American Psychiatric Association. Practice guideline for the treatment of patients with obsessive-compulsive disorder. Am J Psychiatry. (2007) 164 Suppl 7:5–53. 17849776

[20] Marques L LeBlanc NJ Weingarden HM Timpano KR Jenike M Wilhelm S. Barriers to treatment and service utilization in an internet sample of individuals with obsessive-compulsive symptoms. Depress Anxiety. (2010) 27:470–5. doi: 10.1002/da.20694, PMID: 20455248

[21] McKay D Sookman D Neziroglu F Wilhelm S Stein DJ Kyrios M . Efficacy of cognitive-behavioral therapy for obsessive-compulsive disorder. Psychiatry Res. (2015) 227:104–13. doi: 10.1016/j.psychres.2015.02.004 25937054

[22] Williams MT Ching THW. Obsessive-compulsive disorder in ethnoracial minorities: symptoms, barriers to, and considerations for treatment In: Pittenger C, editor. Obsessive-compulsive disorder: Phenomenology, pathophysiology, and treatment. New York: Oxford University Press (2017). 703–14.

[23] Arumugham SS Reddy JY. Augmentation strategies in obsessive-compulsive disorder. Expert Rev Neurother. (2013) 13:187–203. doi: 10.1586/ern.12.160 23368806

[24] Veale D Naismith I Miles S Gledhill LJ Stewart G Hodsoll J. Outcomes for residential or inpatient intensive treatment of obsessive–compulsive disorder: a systematic review and meta-analysis. J Obsessive Compuls Relat Disord. (2016) 8:38–49. doi: 10.1016/j.jocrd.2015.11.005

[25] Gadot R Najera R Hirani S Anand A Storch E Goodman WK . Efficacy of deep brain stimulation for treatment-resistant obsessive-compulsive disorder: systematic review and meta-analysis. J Neurol Neurosurg Psychiatry. (2022) 93:1166–73. doi: 10.1136/jnnp-2021-328738, PMID: 36127157

[26] Dodd S Norman TR Eyre HA Stahl SM Phillips A Carvalho AF . Psilocybin in neuropsychiatry: a review of its pharmacology, safety, and efficacy. CNS Spectr. (2022) 28:416–26. doi: 10.1017/S1092852922000888 35811423

[27] Ling S Ceban F Lui L Lee Y Teopiz KM Rodrigues NB . Molecular mechanisms of psilocybin and implications for the treatment of depression. CNS Drugs. (2022) 36:17–30. doi: 10.1007/s40263-021-00877-y, PMID: 34791625

[28] Studerus E Kometer M Hasler F Vollenweider FX. Acute, subacute and long-term subjective effects of psilocybin in healthy humans: a pooled analysis of experimental studies. J Psychopharmacol. (2011) 25:1434–52. doi: 10.1177/0269881110382466, PMID: 20855349

[29] Griffiths R Richards W Johnson M McCann U Jesse R. Mystical-type experiences occasioned by psilocybin mediate the attribution of personal meaning and spiritual significance 14 months later. J Psychopharmacol. (2008) 22:621–32. doi: 10.1177/0269881108094300, PMID: 18593735 PMC3050654

[30] Hasler F Grimberg U Benz MA Huber T Vollenweider FX. Acute psychological and physiological effects of psilocybin in healthy humans: a double-blind, placebo-controlled dose-effect study. Psychopharmacology. (2004) 172:145–56. doi: 10.1007/s00213-003-1640-6, PMID: 14615876

[31] Tylš F Páleníček T Horáček J. Psilocybin – summary of knowledge and new perspectives. Eur Neuropsychopharmacol. (2014) 24:342–56. doi: 10.1016/j.euroneuro.2013.12.006, PMID: 24444771

[32] Andersen KAA Carhart-Harris R Nutt DJ Erritzoe D. Therapeutic effects of classic serotonergic psychedelics: a systematic review of modern-era clinical studies. Acta Psychiatr Scand. (2021) 143:101–18. doi: 10.1111/acps.13249, PMID: 33125716

[33] Goodwin GM Aaronson ST Alvarez O Arden PC Baker A Bennett JC . Single-dose psilocybin for a treatment-resistant episode of major depression. N Engl J Med. (2022) 387:1637–48. doi: 10.1056/NEJMoa2206443, PMID: 36322843

[34] van Amsterdam J van den Brink W. The therapeutic potential of psilocybin: a systematic review. Expert Opin Drug Saf. (2022) 21:833–40. doi: 10.1080/14740338.2022.2047929, PMID: 35225143

[35] Schneier FR Feusner J Wheaton MG Gomez GJ Cornejo G Naraindas AM . Pilot study of single-dose psilocybin for serotonin reuptake inhibitor-resistant body dysmorphic disorder. J Psychiatr Res. (2023) 161:364–70. doi: 10.1016/j.jpsychires.2023.03.031, PMID: 37004409 PMC10967229

[36] Moreno FA Wiegand CB Taitano EK Delgado PL. Safety, tolerability, and efficacy of psilocybin in 9 patients with obsessive-compulsive disorder. J Clin Psychiatry. (2006) 67:1735–40. doi: 10.4088/jcp.v67n1110, PMID: 17196053

[37] Goodman WK Price LH Rasmussen SA Mazure C Fleischmann RL Hill CL . The Yale-Brown obsessive compulsive scale. I. Development, use, and reliability. Arch Gen Psychiatry. (1989) 46:1006–11. doi: 10.1001/archpsyc.1989.01810110048007 2684084

[38] Goodman WK Price LH Rasmussen SA Mazure C Delgado P Heninger GR . The Yale-Brown obsessive compulsive scale. II Validity. Arch Gen Psychiatry. (1989) 46:1012–6. doi: 10.1001/archpsyc.1989.01810110054008 2510699

[39] Ching THW Grazioplene R Bohner C Kichuk SA DePalmer G D’Amico E . Safety, tolerability, and clinical and neural effects of single-dose psilocybin in obsessive-compulsive disorder: protocol for a randomized, double-blind, placebo-controlled, non-crossover trial. Front Psych. (2023) 14:1178529. doi: 10.3389/fpsyt.2023.1178529, PMID: 37181888 PMC10166878

[40] Kelmendi B Adams T Depalmer G Kichuk S Forte J Grazioplene R . Examining the acute effect of psilocybin in treatment-resistant obsessive-compulsive disorder. ACNP 59^th^ annual meeting: poster session II. Neuropsychopharmacology. (2020) 45:170–277. doi: 10.1038/s41386-020-00891-6, PMID: 33279935 PMC7735200

[41] Barrett FS Johnson MW Griffiths RR. Validation of the revised mystical experience questionnaire in experimental sessions with psilocybin. J Psychopharmacol. (2015) 29:1182–90. doi: 10.1177/0269881115609019, PMID: 26442957 PMC5203697

[42] Davis AK Barrett FS So S Gukasyan N Swift TC Griffiths RR. Development of the psychological insight questionnaire among a sample of people who have consumed psilocybin or LSD. J Psychopharmacol. (2021) 35:437–46. doi: 10.1177/0269881120967878, PMID: 33427007 PMC8056708

[43] Barrett FS Bradstreet MP Leoutsakos JMS Johnson MW Griffiths RR. The challenging experience questionnaire: characterization of challenging experiences with psilocybin mushrooms. J Psychopharmacol. (2016) 30:1279–95. doi: 10.1177/0269881116678781, PMID: 27856683 PMC5549781

[44] Doorley JD Goodman FR Kelso KC Kashdan TB. Psychological flexibility: what we know, what we do not know, and what we think we know. Soc Pers Psychol Compass. (2020) 14:1–11. doi: 10.1111/spc3.12566

[45] Eleftheriou ME Thomas E. Examining the potential synergistic effects between mindfulness training and psychedelic-assisted therapy. Front Psych. (2021) 12:707057. doi: 10.3389/fpsyt.2021.707057, PMID: 34456763 PMC8386240

[46] MacLean KA Johnson MW Griffiths RR. Mystical experiences occasioned by the hallucinogen psilocybin lead to increases in the personality domain of openness. J Psychopharmacol. (2011) 25:1453–61. doi: 10.1177/0269881111420188, PMID: 21956378 PMC3537171

[47] Erritzoe D Roseman L Nour MM MacLean K Kaelen M Nutt DJ . Effects of psilocybin therapy on personality structure. Acta Psychiatr Scand. (2018) 138:368–78. doi: 10.1111/acps.12904, PMID: 29923178 PMC6220878

[48] Blatchford E Bright S Engel L. Tripping over the other: could psychedelics increase empathy? J Psychedelic Stud. (2020) 4:163–70. doi: 10.1556/2054.2020.00136

[49] Hendricks PS. Awe: a putative mechanism underlying the effects of classic psychedelic-assisted psychotherapy. Int Rev Psychiatry. (2018) 30:331–42. doi: 10.1080/09540261.2018.1474185 30260256

[50] Griffiths RR Richards WA McCann U Jesse R. Psilocybin can occasion mystical-type experiences having substantial and sustained personal meaning and spiritual significance. Psychopharmacology. (2006) 187:268–83. doi: 10.1007/s00213-006-0457-5 16826400

[51] Brouwer A Carhart-Harris RL. Pivotal mental states. J Psychopharmacol. (2021) 35:319–52. doi: 10.1177/0269881120959637, PMID: 33174492 PMC8054165

[52] Carhart-Harris RL Roseman L Bolstridge M Demetriou L Pannekoek JN Wall MB . Psilocybin for treatment-resistant depression: fMRI-measured brain mechanisms. Sci Rep. (2017) 7:13187. doi: 10.1038/s41598-017-13282-7, PMID: 29030624 PMC5640601

[53] Cougle JR Fitch KE Jacobson S Lee HJ. A multi-method examination of the role of incompleteness in compulsive checking. J Anxiety Disord. (2013) 27:231–9. doi: 10.1016/j.janxdis.2013.02.003, PMID: 23511304

[54] Garcia-Romeu A Barrett FS Carbonaro TM Johnson MW Griffiths RR. Optimal dosing for psilocybin pharmacotherapy: considering weight-adjusted and fixed dosing approaches. J Psychopharmacol. (2021) 35:353–61. doi: 10.1177/0269881121991822, PMID: 33611977 PMC8056712

[55] Storch EA Rasmussen SA Price LH Larson MJ Murphy TK Goodman WK. Development and psychometric evaluation of the Yale–Brown obsessive-compulsive scale—second edition. Psychol Assess. (2010) 22:223–32. doi: 10.1037/a0018492 20528050

[56] Ching T. Kichuk S. Kelmendi B. Yale program for psychedelic science (YPPS) manual for psilocybin-OCD session monitors for protocol HIC: 2000020355. *PsyArxiv* [Epub ahead of preprint] (2022). doi: 10.31234/osf.io/85s9p

[57] Davis AK Barrett FS May DG Cosimano MP Sepeda ND Johnson MW . Effects of psilocybin-assisted therapy on major depressive disorder: a randomized clinical trial. JAMA Psychiatry. (2021) 78:481–9. doi: 10.1001/jamapsychiatry.2020.3285, PMID: 33146667 PMC7643046

[58] Tolin DF Gilliam C Wootton BM Bowe W Bragdon LB Davis E . Psychometric properties of a structured diagnostic interview for DSM-5 anxiety, mood, and obsessive-compulsive and related disorders. Assessment. (2018) 25:3–13. doi: 10.1177/1073191116638410, PMID: 26988404

[59] First MB Spitzer RL Williams JBW Smith Benjamin L. Structured clinical interview for DSM-5 personality disorders (SCID-5-PD). Washington, DC: American Psychiatric Association (2016).

[60] Babor TF Higgins-Biddle JC Saunders JB Monteiro MG World Health Organization. AUDIT: The alcohol use disorders identification test: Guidelines for use in primary care. 2nd ed. Geneva, Switzerland: World Health Organization (2001).

[61] Berman AH Bergman H Palmstierna T Schlyter F. Evaluation of the drug use disorders identification test (DUDIT) in criminal justice and detoxification settings and in a Swedish population sample. Eur Addict Res. (2005) 11:22–31. doi: 10.1159/000081413 15608468

[62] HEATHERTON TF KOZLOWSKI LT FRECKER RC FAGERSTROM KO. The Fagerström test for nicotine dependence: a revision of the Fagerström tolerance questionnaire. Br J Addict. (1991) 86:1119–27. doi: 10.1111/j.1360-0443.1991.tb01879.x, PMID: 1932883

[63] LECKMAN JF RIDDLE MA HARDIN MT ORT SI SWARTZ KL STEVENSON J . The Yale global tic severity scale: initial testing of a clinician-rated scale of tic severity. J Am Acad Child Adolesc Psychiatry. (1989) 28:566–73. doi: 10.1097/00004583-198907000-00015, PMID: 2768151

[64] Posner K Brent D Lucas C Gould M Stanley B Brown G . Columbia-suicide severity rating scale (C-SSRS). New York: The Research Foundation for Mental Hygiene, Inc (2008).

[65] Ching T. Grazioplene R. Pittenger C. Kelmendi B. Yale program for psychedelic science (YPPS) manual for psilocybin combined with non-directive support in the treatment of OCD. *PsyArxiv* [Epub ahead of preprint] (2023). doi: 10.31234/osf.io/ba42z

[66] Hay A Barthel AL Moskow DM Hofmann SG. Defining and measuring tolerance of uncontrollability. Cogn Ther Res. (2022) 46:259–72. doi: 10.1007/s10608-021-10259-9

[67] Hayes SC Strosahl KD Wilson KG. Acceptance and commitment therapy: The process and practice of mindful change. 2nd ed. New York: Guilford Press (2011).

[68] Kabat-Zinn J. Full catastrophe living: Using the wisdom of your body and mind to face stress, pain, and illness. Revised ed. New York: Bantam Books (2013).

[69] Obsessive Compulsive Cognitions Working Group. Psychometric validation of the obsessive beliefs questionnaire and the interpretation of intrusions inventory: part I. Behav Res Ther. (2003) 41:863–78. doi: 10.1016/s0005-7967(02)00099-2 12880642

[70] Obsessive Compulsive Cognitions Working Group. Psychometric validation of the obsessive beliefs questionnaire and the interpretation of intrusions inventory--part 2: factor analyses and testing of a brief version. Behav Res Ther. (2005) 3:1527–42. doi: 10.1016/j.brat.2004.07.010 16299894

[71] Hartogsohn I. Set and setting, psychedelics and the placebo response: an extra-pharmacological perspective on psychopharmacology. J Psychopharmacol. (2016) 30:1259–67. doi: 10.1177/0269881116677852, PMID: 27852960

[72] Gukasyan N Nayak SM. Psychedelics, placebo effects, and set and setting: insights from common factors theory of psychotherapy. Transcult Psychiatry. (2022) 59:652–64. doi: 10.1177/1363461520983684, PMID: 33499762

[73] U.S. Department of Health and Human Services. Common terminology criteria for adverse events (CTCAE) version 5.0. Bethesda, MD: Cancer Therapy Evaluation Program (2017).

[74] Montgomery SA Åsberg M. A new depression scale designed to be sensitive to change. Br J Psychiatry. (1979) 134:382–9. doi: 10.1192/bjp.134.4.382 444788

[75] Abramowitz JS Deacon BJ Olatunji BO Wheaton MG Berman NC Losardo D . Assessment of obsessive-compulsive symptom dimensions: development and evaluation of the dimensional obsessive-compulsive scale. Psychol Assess. (2010) 22:180–98. doi: 10.1037/a0018260, PMID: 20230164

[76] Jacoby RJ Abramowitz JA Buchholz JL Reuman L Blakey SM. Experiential avoidance in the context of obsessions: development and validation of the acceptance and action questionnaire for obsessions and compulsions. J Obsessive Compuls Relat Disord. (2018) 19:34–43. doi: 10.1016/j.jocrd.2018.07.003

[77] Wegner DM Zanakos S. Chronic thought suppression. J Pers. (1994) 62:615–40. doi: 10.1111/j.1467-6494.1994.tb00311.x 7861307

[78] Gratz KL Roemer L. Multidimensional assessment of emotion regulation and dysregulation: development, factor structure, and initial validation of the difficulties in emotion regulation scale. J Psychopathol Behav. (2004) 26:41–54. doi: 10.1007/s10862-008-9102-4

[79] Chadwick P Hember M Symes J Peters E Kuipers E Dagnan D. Responding mindfully to unpleasant thoughts and images: reliability and validity of the Southampton mindfulness questionnaire (SMQ). Br J Clin Psychol. (2008) 47:451–5. doi: 10.1348/014466508X314891 18573227

[80] Lau M Bishop SR Segal ZV Buis T Anderson ND Carlson L . The Toronto mindfulness scale: development and validation. J Clin Psychol. (2006) 62:1445–67. doi: 10.1002/jclp.20326, PMID: 17019673

[81] Haijen E Kaelen M Roseman L Timmermann C Kettner H Russ S . Predicting responses to psychedelics: a prospective study. Front Pharmacol. (2018) 9:897. doi: 10.3389/fphar.2018.00897, PMID: 30450045 PMC6225734

[82] Nour MM Evans L Nutt D Carhart-Harris RL. Ego-dissolution and psychedelics: validation of the Ego-dissolution inventory (EDI). Front Hum Neurosci. (2016) 10:269. doi: 10.3389/fnhum.2016.00269, PMID: 27378878 PMC4906025

[83] Roseman L Haijen E Idialu-Ikato K Kaelen M Watts R Carhart-Harris R. Emotional breakthrough and psychedelics: validation of the emotional breakthrough inventory. J Psychopharmacol. (2019) 33:1076–87. doi: 10.1177/0269881119855974 31294673

[84] Neff KD. Development and validation of a scale to measure self-compassion. Self Identity. (2003) 2:223–50. doi: 10.1080/15298860390209035

[85] Gosling SD Rentfrow PJ Swann WB. A very brief measure of the big-five personality domains. J Res Pers. (2003) 37:504–28. doi: 10.1016/S0092-6566(03)00046-1

[86] Stevanovic D. Quality of life enjoyment and satisfaction questionnaire-short form for quality of life assessments in clinical practice: a psychometric study. J Psychiatr Ment Health Nurs. (2011) 18:744–50. doi: 10.1111/j.1365-2850.2011.01735.x 21896118

[87] Ustun TB Kostanjesek N Chatterji S Rehm J World Health Organization. Measuring health and disability: Manual for WHO disability assessment schedule (WHODAS 2.0). Geneva, Switzerland: WHO (2010).

[88] Hatcher RL Gillaspy JA. Development and validation of a revised short version of the working Alliance inventory. Psychother Res. (2006) 16:12–25. doi: 10.1080/1050330050035250

[89] Younger J Gandhi V Hubbard E Mackey S. Development of the Stanford expectations of treatment scale (SETS): a tool for measuring patient outcome expectancy in clinical trials. Clin Trials. (2012) 9:767–76. doi: 10.1177/1740774512465064 23169874

[90] Worthington RL Dillon FR. The theoretical orientation profile scale-revised: a validation study. Meas Eval Couns Dev. (2003) *36*:95–105. doi: 10.1080/07481756.2003.12069085

[91] Tendler A Sisko E Barnea-Ygael N Zangen A Storch EA. A method to provoke obsessive compulsive symptoms for basic research and clinical interventions. Front Psych. (2019) 10:0814. doi: 10.3389/fpsyt.2019.00814, PMID: 31824345 PMC6882501

[92] Smith JA Flowers P Larkin M. Interpretative phenomenological analysis: Theory, research, practice. London, UK: SAGE (2009).

