# Corrigendum: Safety, feasibility, tolerability, and clinical effects of repeated psilocybin dosing combined with non-directive support in the treatment of obsessive-compulsive disorder: protocol for a randomized, waitlist-controlled trial with blinded ratings

**DOI:** 10.3389/fpsyt.2024.1372373

**Published:** 2024-02-16

**Authors:** Terence H. W. Ching, Lucia Amoroso, Calvin Bohner, Elizabeth D'Amico, Jeffrey Eilbott, Tara Entezar, Madison Fitzpatrick, Geena Fram, Rachael Grazioplene, Jamila Hokanson, Anastasia Jankovsky, Stephen A. Kichuk, Bradford Martins, Prerana Patel, Henry Schaer, Sarah Shnayder, Chelsea Witherow, Christopher Pittenger, Benjamin Kelmendi

**Affiliations:** ^1^ Department of Psychiatry, Yale University School of Medicine, New Haven, CT, United States; ^2^ Department of Psychology, Yale University, New Haven, CT, United States; ^3^ Center for Brain and Mind Health, Yale University School of Medicine, New Haven, CT, United States; ^4^ Child Study Center, Yale University School of Medicine, New Haven, CT, United States

**Keywords:** obsessive-compulsive disorder, psilocybin, psychedelic, non-directive support, waitlist, treatment, mental health, adult psychiatry

**Error in Author List**

In the published article, there was an error in the author list, and Anastasia Jankovsky was erroneously excluded. The corrected author list appears below.

Terence H. W. Ching,^1^ Lucia Amoroso,^1^ Calvin Bohner,^1^ Elizabeth D'Amico,^1^ Jeffrey Eilbott,^1^ Tara Entezar,^1^ Madison Fitzpatrick,^1^ Geena Fram,^1^ Rachael Grazioplene,^1^ Jamila Hokanson,^1^ Anastasia Jankovsky,^1^ Stephen A. Kichuk,^1^ Bradford Martins,^1^ Prerana Patel,^1^ Henry Schaer,^1^ Sarah Shnayder,^1^ Chelsea Witherow,^1^ Christopher Pittenger,^1-4^ Benjamin Kelmendi^1^

The authors apologize for this error and state that this does not change the scientific conclusions of the article in any way. The original article has been updated.

